# A systematic review of hormone levels, biomarkers of cellular injury and oxidative stress in multi-stressor military field training exercises

**DOI:** 10.20945/2359-3997000000443

**Published:** 2022-03-16

**Authors:** Filipe Brasil e Silva, Mario Vaisman, Thalita Ponce, Thiago Ramos de Barros, Camila Brasil e Silva, Verônica Pinto Salerno, Míriam Raquel Meira Mainenti

**Affiliations:** 1 Escola de Educação Física do Exército Rio de Janeiro RJ Brasil Escola de Educação Física do Exército, Rio de Janeiro, RJ, Brasil; 2 Universidade Federal do Rio de Janeiro Faculdade de Medicina Departamento de Medicina Interna – Endocrinologia Rio de Janeiro RJ Brasil Departamento de Medicina Interna – Endocrinologia, Faculdade de Medicina, Universidade Federal do Rio de Janeiro, Rio de Janeiro, RJ, Brasil; 3 Universidade Federal do Rio de Janeiro Escola de Educação Física e Desportos Rio de Janeiro RJ Brasil Escola de Educação Física e Desportos, Universidade Federal do Rio de Janeiro, Rio de Janeiro, RJ, Brasil; 4 Academia de Bombeiro Militar Dom Pedro II Rio de Janeiro RJ Brasil Academia de Bombeiro Militar Dom Pedro II, Rio de Janeiro, RJ, Brasil; 5 Companhia de Comando da 4ª Brigada de Infantaria Leve de Montanha Exército Brasileiro Juiz de Fora MG Brasil Companhia de Comando da 4ª Brigada de Infantaria Leve de Montanha – Exército Brasileiro, Juiz de Fora, MG, Brasil

**Keywords:** Military training, physical effort, food deprivation, endocrinology, oxidative damage

## Abstract

The fundamental objective of military field training exercises (FTX) is to prepare military personnel for real-life operations through simulated scenarios. These training sessions often require extreme physical efforts with prolonged, high-intensity exercises that can be combined with food restrictions and partial, or total, sleep deprivation. Such conditions can compromise an individual’s physical performance and cause tissue damage, thus affecting their health. This study aimed to perform a systematic review of the literature to identify studies that measured the changes in hormone levels and biomarkers of cellular injury and oxidative stress resulting from FTX with high levels of energy expenditure combined with food and sleep restrictions. PubMed and the Scopus database were searched for articles that combined physical effort/food restriction/sleep deprivation with military training. The initial database search identified 158 articles that were reduced to 18 after confirmation. Significant reductions were reported in thyroid hormones, T3, T4, and anabolic hormones such as testosterone, insulin and androstenedione. An exception for GH was found, which increased throughout FTX. Less distinct responses to FTX were observed with cortisol, TSH and LH. The presence of biomarkers for cellular damage (myoglobin, TNF, and CRP) and increased immune response activities were also described. The scarcity of information on oxidative stress, analyses of cellular injury and biomarkers of inflammatory responses warrants the future study of these topics, which could be helpful in facilitating the safe and effective physical preparations of the members of the armed forces.

## INTRODUCTION

Military field training exercises (FTX) encompasses periodical and coordinated tactical operations with deployed units to prepare personnel for active duty. Designed to reflect the real-life challenges the armed services may encounter, the conditions simulated in an FTX involves high-intensity, prolonged, and extreme physical efforts together with food restrictions, psychological stresses, temperature variations, and sleep deprivation ( 1 , 2 ). When military personnel experience physical exertion and inadequate rest periods, such scenarios typically leads to cellular damage, impaired muscle strength, and a myriad of physical injuries ( 3 ). The literature points out that these consequences can be inferred by elevations in the serum biomarkers of muscular damage, hormonal imbalances, and the presence of elevated levels of oxidative agents, all of which can negatively impact military operational readiness ( 1 , 3 , 4 ).

Muscle damage can be indicated by increases in the serum levels of specific biomarkers. These can either be related to the rupture of muscle fibers during and after training sessions, with the posterior release of its cell contents into the blood ( 5 ), or to other metabolites that originate from different organs, such as the kidneys, liver, pancreas, and brain ( 3 , 4 ). Among the measured biomarkers are alanine transaminase (ALT), aspartate transaminase (AST), creatine kinase (CK), lactate dehydrogenase (LDH), myoglobin (Mb), C-reactive protein (CRP), and alpha-1-acid glycoprotein (AGPA) ( 3 , 4 ).

Physical activity also releases the production of free radicals that are related to the intensity of a workload on metabolic processes ( 3 ). These free radicals are reactive oxygen species (ROS) containing unpaired electrons that are generated during the ordinary course of the aerobic energy pathway when all the electrons do not reach the end of the chain ( 6 ). When unchallenged by the protection system, excess ROS can induce an imbalance called oxidative stress. This phenomenon oxidizes biomolecules that can cause cell damage and a loss of cellular function, ultimately leading to tissue and organ injury ( 7 ). However, up to a specific limit, the release of ROS can be beneficial since they can act as signaling molecules to stimulate adaptations related to physical training exercises, principally through increases in antioxidant capacities. This, in turn, provides improved resistance to oxidative stress, and the organism can protect cells normally affected by oxidative stress and prevent its deleterious effects ( 8 ).

Food restrictions and long durations of high-intensity “endurance-like” training as experienced during military field activities are contributing factors to physical damage. While endurance athletes have greater control over the repositioning of macronutrients, military units performing FTX might be limited to replenishing themselves on strict schedules or only when the opportunity allows. These limits on food consumption usually causes energy deficits that leads to more severe muscular damage and changes in serum hormone levels, such as testosterone, growth hormone (GH), insulin, cortisol, T3, T4, and TSH. Physical distress, sleep deprivation and food restrictions during FTX ( 9 ) ultimately causes the undesirable failure of individual physical performance ( 10 ), which, for military personnel, can translate as a failure to complete the training or the real-world mission.

Inadequate sleep and resting periods observed during FTX, along with an increase in free radicals and inflammatory mediators, can increase oxidative stress and, ultimately, result in cellular damage. Sleep deprivation impairs the mechanisms that adjust the metabolism and immunological systems and the restoration of energy storage in biological systems, such as muscle glycogen ( 2 ), which can impact the muscle recovery process required to improve and ensure maintained operational readiness.

Considering this, it is possible to assume that the triad of intense physical demand/food restriction/sleep deprivation typically experienced during military field training exercises (FTX) intensifies the physiological stress of the participants, which can, eventually, result in adverse clinical outcomes. A commonly occurring condition is rhabdomyolysis, which is the process of massive cell destruction wherein cell contents are released into the bloodstream associated to relevant clinical conditions such as hyperphosphatemia, hypercalcemia, disseminated intravascular coagulation, compartment syndrome, and cardiac arrhythmias ( 11 , 12 ).

Although a variety of biomarkers from individuals who have participated in FTX sessions have been previously studied ( 1 – 4 , 10 , 13 ), a compilation of these data would be convenient, especially considering the potential effects of the three abovementioned factors applied together. It is important to identify and understand the individual effects of each of the three factors, yet it is imperative to analyze the simultaneous effects because doing so better reflects the actual scenarios of most military operations that are being simulated by the FTX. This motivates the current work that aims to compile the most frequent physiological changes in serum biomarkers of hormone imbalances, oxidative stress and cellular injury measured as the result of a military field training exercise. The recent expansion into various countries regarding studies on military personnel that involve military/civilian/academic institution partnerships is specifically focused on ensuring effective and safe military training programs. This is the first systematic review of the literature regarding changes in hormone and biochemical levels due to FTX that can support researchers in diverse areas, such as endocrinology, metabolism and exercise, as they contribute to this area of study. Included are studies involving both male and female members of the military, which both acknowledges the recent increase in female recruitment in many armed forces and highlights the need to consider both sexes. We anticipate that the results of this review will enable a more robust prediction of the impact of a participant’s health condition on the outcomes of training and, consequently, provide relevant tools to guide the planning and applications of future military exercises.

## METHODS

Searches were performed on PubMed and Scopus databases to select articles published before December 1, 2020 using the terms: “military training” OR “military exercise” OR “physical exercise” OR “high intensity exercise” OR “caloric cost” OR “caloric expenditure” OR “energy cost” OR “energy expenditure” OR endurance OR exhaustion) AND (“caloric restriction” OR “calorie restriction” OR “food restriction” OR “food intake” OR “food consumption” OR “energy intake” OR “fasting”) AND (“sleep restriction” OR “sleep deprivation” OR “sleep shortage” OR “rest deprivation”) AND (“cell injury” OR “cell damage” OR “muscle injury” OR “muscle damage” OR biomarker OR “oxidative stress” OR hormonal OR hormone OR antioxidant OR stress OR oxidant).

The search aimed to select publications that involved changes in biomarkers resulting from the joint interventions of physical effort/food restriction/sleep deprivation in young adult military units under training. Review studies and those using animal models were excluded. The remaining articles were independently reviewed by two members of the study (FB and TB) for inclusion or exclusion. First, the titles and abstracts were compared to exclude duplicate articles and analyzed for the elegibility criteria. Then, each was read in its entirety to scrutinize the inclusion and exclusion criteria. Those with relevant citations had their primary references hand searched and analyzed. Any discrepancies were resolved by a third reviewer (TP). The resulting process was to list the publications that fulfilled all the inclusion criteria and served the present review’s primary objective.

Five different domains were evaluated to assess the quality and risk of bias of the selected articles according to the *Cochrane Risk of Bias Tool* ( 14 ), which is not limited to randomized controlled trials (RCTs): s *election bias:* related to subject allocation methods and its blinding. *Performance bias:* related to blinding both the subjects and the researchers on the former’s allocation data. *Detection bias:* related to the blinding of the evaluators about the subject’s allocation data. *Attrition bias:* related to lost subjects to specific results or unavailability of result’s data. *Reporting bias:* related to the chances of presenting only convenient or relevant results. Each domain constituted an uncertain (U), low (L), or high (H) risk of bias. The articles’ evaluation was conservative, and a high risk of bias was related to the absence of records or uncertainty in the observation of the quality domains. Two researchers conducted the process (FB and TB), and a third one (CB) analyzed any discrepancies.

An illustrative flowchart was designed to expose the study selection methodology. In addition, a table presents the summarized results, containing the studies’ main features and the biomarkers analyzed in each article. The entire systematic review process was based on PRISMA ( *Preferred Reporting Items for Systematic Reviews and Meta-Analyses* ) recommendations ( 15 ).

## RESULTS

**Figure 1:**
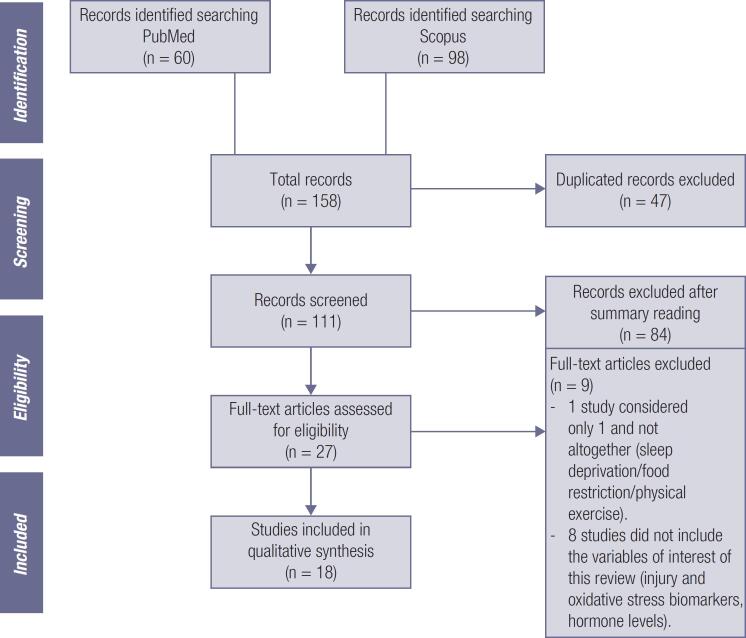
PRISMA flowchart of the study selection process.

The literary search of the Scopus and PubMed databases retrieved 158 articles. After thoroughly analyzing the inclusion and exclusion criteria, 18 articles were identified that met the defined criteria. The flowchart in Figure 1 presents a summary of the selection process.

The results from the evaluation of the risk of bias for each article are shown in Table 1 . Most of the articles reviewed presented low risk of bias. The “performance” domain was the one which presented the higher prevalence of high risk of bias.

**Table 1:** Study risk of bias assessment based on the Cochrane Risk of Bias Tool ( 14 )

Reference	Year	Selection	Performance	Detection	Attrition	Report
Opstad *et al.* ( 16 )	1980	L	L	L	H	L
Opstad, Aakvaag ( 17 )	1981	L	H	L	L	L
Opstad, Aakvaag ( 18 )	1982	L	H	U	L	L
Oektedalen *et al.* ( 19 )	1982	L	H	H	L	L
Opstad, Aakvaag ( 20 )	1983	L	L	U	L	L
Oektedalen *et al.* ( 21 )	1983	L	L	U	L	L
Øktedalen *et al.* ( 22 )	1983	L	L	U	L	L
Opstad *et al.* ( 23 )	1984	L	L	L	L	L
Guezennec *et al.* ( 24 )	1994	L	H	U	L	L
Opstad *et al.* ( 25 )	1994	L	L	L	L	L
Nindl *et al.* ( 26 )	2003	L	L	L	L	L
Gomez-Merino *et al.* ( 27 )	2005	L	L	L	H	H
Nindl *et al.* ( 9 )	2006	L	L	L	L	L
Booth *et al.* ( 28 )	2006	L	L	L	L	L
Gundersen *et al.* ( 29 )	2006	L	L	L	L	L
Nindl *et al.* ( 30 )	2007	U	L	L	L	L
Alemany *et al.* ( 31 )	2008	L	H	L	U	L
Henning *et al.* ( 32 )	2014	L	L	L	H	L

H: high risk of bias; L: low risk of bias; U: uncertain risk of bias.

A short description of each of the 18 articles reviewed is presented in Table 2 , in chronological order, with their features and results. The different variables of interest were recorded from two distinct moments, before and after the triad of exercise conditions, which allowed for comparison of the results.

**Table 2:** Reviewed articles, interventions and results

Reference	Year	Sample	Intervention	Measured variables/effect
Opstad *et al.* ( 16 )	1980	n=11, men Age: $\bar{\text{x}}$ 23.7 years TBM: NR	Physical activity: 4.5 days of military training Food intake: 1,500 kcal/day Sleep: 1-2 hours total during training Actual FTX Temperature condition: 25 °C – day; 5 °C - night	↑TSH ↓T4↓T3 ↔Insulin ↑GH ↑prolactin ↑dopamine ↑epinephrine ↑norepinephrine
Opstad, Aakvaag ( 17 )	1981	n=11, men Age: $\bar{\text{x}}$ 22.9 years TBM: NR	Physical activity: 5 days of military training Food intake: G1: 6,400 kcal/day G2: 1,500 kcal/day Sleep: 2 hours total during training Actual FTX Temperature condition: 25 °C (max, day); 5-10 °C (min, night)	G1: ↓T3 ↓T4 ↓TSH ↑GH ↓↓Cortisol ↓ Insulin G2: ↓↓T3 ↓T4 ↓↓TSH ↑↑GH ↓Cortisol ↓↓Insulin
Opstad, Aakvaag ( 18 )	1982	n=11, men Age: $\bar{\text{x}}$ 22.9 years TBM: NR	Physical activity: 5 days of military training Food intake G1: 6,400 kcal/day G2: 1,500 kcal/day Sleep: 2 hours total during training Actual FTX Temperature condition: ND	G1: ↓Estradiol ↓prolactin ↓testosterone G2: ↓Estradiol ↓prolactin ↓testosterone
Oektedalen *et al.* ( 19 )	1982	n=24, men Age: 23.5 ± 2.5 years TBM: NR	Physical activity: 5 days of military training Food intake and sleep: G1: 1,500 kcal/day and 1-2 hours total during training G2: 6,300 kcal/day and 1-2 hours total during training, G3: 1,500 kcal/day and 3 hours daily Actual FTX Temperature condition: ND	G1: ↑secretin G2: ↑secretin G3: ↑secretin
Opstad, Aakvaag ( 20 )	1983	n=17, men Age: $\bar{\text{x}}$ 24.3 years TBM: NR	Physical activity: 5 days of military training Food intake: 1,500 kcal/day Sleep: G1: 1-3 hours total during training G2: 3 hours daily Actual FTX Temperature condition: 25-30 °C (max, day); 5-10 °C (min, night)	G1: ↑TSH ↓T3 ↓T4 ↓↓DHT ↓↓androstenedione ↓↓testosterone ↑GH ↓prolactin ↓↓cortisol ↓LH ↓dopamine ↓epinephrine ↓↓norepinephrine G2: ↑TSH ↓T3 ↓T4 ↓DHT ↓androstenedione ↓testosterone ↑GH ↓prolactin ↓cortisol ↓↓LH ↓dopamine ↓epinephrine ↓norepinephrine
Oektedalen *et al.* ( 21 )	1983	n=20, men Age: 25 ± 5 years TBM: NR	Physical activity: 5 days of military training Food intake: ND Sleep: 6-7 hours total during training G1: had 1 g cimetidine intake daily G2: had placebo intake Actual FTX Temperature condition: ND	G1: ↑secretin ↑VIP ↔gastrin ↑ stomach acid ↑pentagastrin ↔glucose G2: ↑↑secretin ↑VIP ↑gastrin ↑↑ stomach acid ↑↑pentagastrin ↓glucose
Øktedalen *et al.* ( 22 )	1983	n=24, men Age: 23.5 ± 2.5 years TBM: NR	Physical activity: 5 days of military training Food intake and sleep: G1: 1,500 kcal/day and 1-2 hours total during training G2: 6,500 kcal/day e 1-2 hours total during training G3: 1,500 kcal/day e 3 hours daily G4: 5 days of fasting (did not take part in the training sessions) Actual FTX Temperature condition: ND	G1: ↔GIP ↓insulin G2: ↔GIP ↓insulin G3: ↔GIP ↓insulin G4: ↔GIP ↓insulin
Opstad *et al.* ( 23 )	1984	n=24, men Age: 24 ± 3 years TBM: NR	Physical activity: 5 days of military training Food intake and sleep: G1: 1,200 kcal/day and 1-3 hours total during training G2: 8,000 kcal/day and 1-3 hours total during training G3: 1,200 kcal/day and 3 hours daily Actual FTX Temperature condition: approximately 20 °C	G1: ↓TSH ↓T3 ↓T4 ↓↓FT4 ↔TBG ↑rT3 G2: ↓TSH ↑T3 ↑T4 ↓FT4 ↑TBG ↔rT3 G3: ↓TSH ↓T3 ↓T4 ↓↓FT4 ↔TBG ↑rT3
Guezennec *et al.* ( 24 )	1994	n=27, men Age: NR TBM: NR	Physical activity: 4 days of military training Food intake G1: 1,800 kcal/day G2: 3,200 kcal/day G3: 4,200 kcal/day Sleep: 3-4 hours daily Actual FTX Temperature condition: 18-25 °C	G1: ↓insulin ↓↓testosterone ↓glucose ↑↑FFA ↑myoglobin G2: ↓insulin ↓testosterone ↔glucose ↑FFA ↑myoglobin G3: ↓insulin ↓testosterone ↔glucose ↑FFA ↑myoglobin
Opstad *et al.* ( 25 )	1994	n=31, men Age: 28 ± 2 years TBM: NR	Physical activity: 6 days of military training Food intake: ND Sleep: 1-3 hours total during training Actual FTX Temperature condition: ND	↔ ANP
Nindl *et al.* ( 26 )	2003	n=12, men Age: 23 ± 1 years TBM: 85 ± 4 kg	Physical activity: 3 days of military training Food intake: ND Sleep: 1 hour daily Simulated FTX Temperature condition: ND	↓total IGF-1 ↓free IGF-1 ↓nonternary IGF-I ↓IGFBP-1 ↓IGFBP-3 ↑ferritin ↑FFA ↑glycerol ↑glucose ↑hydroxybutyrate ↓prealbumin
Gomez-Merino *et al.* ( 27 )	2005	n=21, men Age: 21 ± 2 years TBM: NR	Physical activity: 3 weeks and 5 days of military training Food intake: ND Sleep: ND Actual FTX Temperature condition: 12-25 °C	↓cortisol ↓testosterone ↓leptin ↑DHEA-S ↑IL-6 ↑dopamine ↑lymphocytes ↑leukocytes ↑neutrophils ↑platelets ↑monocytes ↑white-cell count ↑ NK ↑CD4+ ↑CD3+ ↑CD8+
Nindl *et al.* ( 9 )	2006	n=10, men Age: 22 ± 3 years TBM: 87 ± 8 kg	Physical activity: 4 days of military training Food intake: 2,800 kcal/day Sleep: 2 hours daily Simulated FTX Temperature condition: ND	↑LH ↑GH ↔leptin ↓testosterone ↓total IGF-1 ↓free IGF-1 ↓IGFBP-3 ↑IGFBP-1 ↑FFA ↔glucose
Booth *et al.* ( 28 )	2006	n=38, men Age: $\bar{\text{x}}$ 22 years TBM: $\bar{\text{x}}$ 78.8 kg n=5, women Age: $\bar{\text{x}}$ 29.5 years TBM: $\bar{\text{x}}$ 64.5 kg	Physical activity: 45 days of military training Food intake: ND Sleep: ND Actual FTX Temperature condition: ND	Men: ↑cortisol ↓testosterone ↑CRP ↑TNF ↓lymphocytes ↓neutrophils ↓hemoglobin ↓ferritin Women: ↑cortisol ↓testosterone ↑CRP ↑TNF ↓lymphocytes ↓neutrophils ↓hemoglobin ↓ferritin
Gundersen *et al.* ( 29 )	2006	n=8, men Idade: 25.8 ± 0.9 years TBM: 80 ± 3.7 kg	Physical activity: 7 days of military training Food intake: 300-800 kcal/day Sleep: 1 hour daily Actual FTX Temperature condition: ND	↓leptin ↑cortisol ↑IL-1 ↑IL-6 ↑CRP ↑TNF-alpha ↑IL-beta ↑MMP-9 ↔TIMP-1 ↑leukocytes ↑monocytes ↓hemoglobin ↓hematocrit
Nindl *et al.* ( 30 )	2007	n=34, men Age: 24 ± 3 years TBM: 82.3 ± 7.8 kg	Physical activity: 8 days of military training Food intake: 1,000 a 1,500 kcal/day Sleep: 4 hours daily Actual FTX Temperature condition: ND	↑T4 ↑GH ↓testosterone ↓leptin IGF-1 system: ↓ IGF-1 total/IGFBP-2 ↓ IGF-1total/IGFBP-3 ↓ IGF-I total/ALS ↓IGFBP-3/IGFBP-2 ↓ALS/IGFBP-2 ↔ALS/IGFBP-3 ↑ferritin
Alemany *et al.* ( 31 )	2008	n=34, men Age: 24.5 ± 0.3 years TBM: 83 ± 1.4 kg	Physical activity: 8 days of military training Food intake G1: protein intake of 0.5 g/kg of body mass daily G2: protein intake of 0.9 g/kg of body mass daily Sleep: ND Actual FTX Temperature condition: 19.3-29.6 °C	G1: ↓total IGF-1↓free IGF-1 ↓nonternary IGF-1 ↓IGFBP-3 ↑ALS ↓testosterone ↑↑SHBG ↓↓DHEA ↑DHEA-S G2: ↓total IGF-1 ↓free IGF-1 ↓nonternary IGF-1 ↓IGFBP-3 ↑ALS ↓testosterone ↑SHBG ↓↓DHEA ↑DHEA-S
Henning *et al.* ( 32 )	2014	n=23, men Age: 23 ± 2.8 years TBM: 81 ± 9.6 kg	Physical activity: 8 weeks of military training Food intake: 2,200 kcal/day Sleep: 0-5 hours daily Actual FTX Temperature condition: ND	↓T3 ↑TSH ↔T4 ↓Testosterone ↑SHBG ↔cortisol ↓BDNF ↓total IGF-1 ↓free IGF-1 ↑↑IGFBP-1 ↑↑IGFBP-2 ↑IGFBP-3 ↓IGFBP-6 ↑IL-4 ↑IL-6 ↑IL-8 ↔IL-1 ↔IL-10 ↔TNF ↔CRP

ALS: acetolactate synthase; ANP: atrial natriuretic peptide; BDNF: brain-derived neurotrophic factor; CD+: glycoprotein; CRP: c-reactive protein; DHEA: dehydroepiandrosterone; DHEA-S: dehydroepiandrosterone sulfate; DHT: dihydrotestosterone; FFA: fatty free acids; FT4: free thyroxine; FTX: military field training exercise; G1: Group 1; G2: Group 2; G3: Group 3; G4: Group 4; GH: growth hormone; GIP: gastric inhibitory peptide; IGF-1: insulin-like growth Factor 1; IGFBP insulin-like growth factor binding protein; IL: interleukin; LH: luteinizing hormone; max: maximum; min: minimum; MMP-9: matrix metallopeptidase 9; ND: not described; NK cells: natural-killer cells; NR: not recorded; rT3: reverse triiodothyronine; SHBG: sex hormone-binding globulin; T3: triiodothyronine; T4: thyroxine; TBG: thyroxine binding globulin; TBM: total body mass; TNF: tumor necrosis factor; TSH: thyroid-stimulating hormone; TIMP-I: tissue inhibitor of metalloproteinases I; VIP: vasoactive intestinal peptide; ↔ = no significant change; ↑ = significant rise; ↑↑ = prominent significant rise; ↓ = significant reduction; ↓↓ = prominent significant reduction.

Overall, the vast majority of articles reviewed involved male participants between 20 and 30 years of age. Only one study included female military members ( 28 ). The sample sizes of the study groups ranged from five to thirty-eight subjects (5 ≤ n ≤ 38), and their body masses ranged between 71.4 kg and 95 kg, which we obtained from a subset of seven articles ( 9 , 26 , 28 – 32 ), all of which included only male participants. The mean total body mass of the female participants was 64.5 kg ( 28 ).

As selected by the criteria, all publications involved the combination of intense physical activity, food restriction, and sleep deprivation, with most studies occurring over 3 to 8 days. Three studies were performed during a longer period: 3 weeks and 5 days ( 27 ), 45 days ( 28 ), and 8 weeks ( 32 ). Food consumption during training ranged from 300 to 8,000 kcal/day, while the periods of time allotted to sleeping ranged from 0 to 3 hours per day or per session. In eight studies ( 16 – 23 ), these variables were handled by dividing the subjects into groups, with a particular calorie intake and sleep regimen for each. Most studies (89%) were conducted during an actual FTX as opposed to a simulated FTX (two articles). The temperature conditions were similar among the seven studies (39%), which reported a maximum daily temperature between 25-30 °C.

Five articles analyzed TSH levels ( 16 , 17 , 20 , 23 , 32 ). Three reported an increase (60%), and two reported a decrease (40%) after FTX. In the five studies that analyzed T3 hormone, all the studies reported that T3 levels decreased except for a group in one study that received a daily calorie intake of 8,000 kcal ( 23 ). The T4 hormone was measured in six publications, with four (66%) studies reporting lower levels after FTX, one study (16%) showing no change and one study (16%) showing higher levels. The last report came from the same study which showed an increase in T3 ( 23 ) that was only for the subgroup that received the 8,000-kcal diet. In the study that showed unchanged levels of T4, the participants underwent eight weeks of military training, which was the most prolonged intervention found in this review ( 31 ). The other measured thyroid hormones showed no trends and are presented in Table 2 .

Anabolic hormone levels were documented in a number of studies. Insulin was analyzed in four publications, and its level was reported as lower in three (75%) and unchanged in one (25%). GH was studied in five articles and showed an increase in all (100%) of them. In the nine studies that evaluated the level of testosterone after FTX, all (100%) of the studies reported a decrease. Only two studies reported on LH with a different response in each. The gonadal hormone levels and hypothalamus-pituitary-gonadal axis-related hormone levels (estradiol, DHEA, DHT, and androstenedione) all presented lower levels in the articles reviewed, however the levels of DHEA-S showed an increase. Four studies analyzed the insulin-like growth factor fractions (both free and total), and each reported lower levels after the training program. An inconsistent response in the levels of the IGF binding proteins was observed. IGFBP-I levels rose in two studies (66%) and decreased in one (33%) study. IGFBP-2 and IGFBP-3 levels appeared to be augmented, and one record of IGFBP-6 showed a decrease in its concentration.

The catabolic hormone, cortisol, presented inconsistent results: two studies showed that cortisol levels increased (28%), four studies showed a decrease (56%), and one study showed unchanged levels (14%). Prolactin appeared on three occasions, and it was decreased in two (66%) and increased in one (33%). Secretin increased in both studies where it was reported (100%), while leptin levels were unchanged in one study (25%) and decreased in three (75%). Other hormone levels are presented in Table 2 .

Two studies reported on inflammatory mediators, namely lymphocytes and neutrophils, with one study reporting an increase and a reported decrease in the other. Two studies reported an increase in monocytes and leukocytes (100%). One publication assessed the white cell count (NK cells, CD3+, CD4+, and CD8+) and observed an increase in all four (100%). Another study showed a decrease in hematocrit levels (100%). C-reactive protein and TNF appeared in three articles, two reported an increase (66%) and the other reported a decrease (33%). For IL-6, three articles reported a solid increase (100%), while IL-2 levels were reported to be higher in one study and unchanged in another (50-50%). Other interleukins were measured and are shown in Table 2 .

Few studies that included the triad of conditions also assessed the biomarkers of cellular injury. An increase of myoglobin was found in one (100%) study, and two records (100%) reported a decrease of hemoglobin. Proteins, such as SHBG and BDNF, were also found during our review, with a reported increase of the former in two articles and a decrease of BDNF in one (100%) study. Finally, this review did not retrieve a single study that considered the three interventions and biomarkers of oxidative stress.

## DISCUSSION

The combined scenario including prolonged physical exertion, food restriction, and sleep deprivation is directly related to physiological disturbances ( 9 ). The present review aimed to analyze and observe the role of this triad of interventions on hormone imbalances and biomarkers of cellular injury and oxidative stress in military field training exercises (FTX). Some studies manipulated the three factors, especially regarding food consumption and sleep conditions, presenting multiple groups. However, since the aim of the present review was to observe the combined effect, we did not discuss their individual effects in this paper.

Our results suggests decreases in thyroid and anabolic hormone levels (except for increased levels of GH) and increases in inflammatory mediators and biomarkers for cellular injury, although the last two variables were infrequently studied by the time of this review. Additionally, there is disagreement regarding the behavior of some hormones with the triad of interventions, especially regarding the levels of TSH, LH, and cortisol, and there was no information on the biomarkers for oxidative stress that considered the simultaneous application of the three different interventions.

The impact of the triad of interventions on various biomarkers might explain the changes often observed in the body composition of participants after FTX. Considering the role of thyroid hormones in protein synthesis and the regulation of cellular metabolism, their decreased levels observed after military exercise might be one of the reasons behind diminished fat free mass, fat mass, and total body mass ( 16 , 33 , 34 ). Higher levels of GH can further influence these changes in body composition. This hormone directs lipolysis as the preferred energy storage mobilization pathway ( 32 ), and an energy deficit induces glucose and muscular glycogen consumption. Evidence for higher lipolytic activity has been presented by Guezennec and cols. ( 24 ) and Nindl and cols. ( 9 , 35 ), who reported higher levels of glycerol and free fatty acid (FFA) serum levels. Other indications for demands on lipid metabolism are the observed increases in hydroxybutyrate, which is related to fatty acid metabolism ( 35 ), and a decrease in the levels of leptin, which are related to the available quantities of adipose tissue.

The variations in insulin-like growth Factor 1 (IGF-1) and its related counterparts reported after FTX could contribute to the lower anabolic metabolism rates seen in this review. IGF-1 secretion occurs in the liver and is regulated by GH levels. Its circulating forms of plasma exert control over some metabolic pathways that permits the use of IGF-1 as an adequate biomarker of tissue remodeling, nutritional state, and metabolic adaptations ( 35 ). In the present review, we noticed that the intervention triad in FTX led to a reduction in free and total IGF-1 levels, even though levels of GH appeared elevated ( 9 , 30 – 32 , 35 ). This phenomenon suggests resistance in the liver to the activities of GH in scenarios of stress and low-calorie availability, which could impair the secretion of IGF-1 ( 29 , 36 ). DeBoer and cols. ( 36 ) suggests that inflammatory mediators influence these limitations in hepatic function, a hypothesis supported by the abundance of studies presenting increases in these biomarkers during our review ( 27 – 29 , 32 ). Additionally, the increase of IGF binding protein 1, a well-known inhibitor of IGF-1 ( 37 ), would most likely sustain the decreased anabolism rates even further.

Our study verified the direct impact of anabolism via the decrease observed in anabolic hormones. All the articles reviewed presented evidence for lower quantities of circulating testosterone, estradiol, dihydrotestosterone (DHT), insulin, and androstenedione ( 9 , 18 , 20 , 24 , 27 , 28 , 30 – 32 ). Opstad and Aakvaag ( 18 ) also noted a consistent decrease in these levels in all subjects despite providing a high-calorie diet to participants. This observation appears to result from the insufficient availability of energy combined with extreme physical exertion ( 24 ), which ultimately leads to the loss of the capacity to recover, loss of muscle repair and impaired physical performance, which are undesirable consequences for military personnel in training and in actual operations.

The reported changes in cortisol levels were inconsistent between the articles reviewed. The levels appeared to be lower when the FTX included a more limited calorie intake ( 28 ) or a longer period of training, 45 days ( 27 ). Planning FTX programs for shorter durations of time would diminish the training volume but could be a strategy to avoid a loss of performance. However, if greater physical intensity is the goal of FTX, then instructors should be informed that possible adverse outcomes could become more frequent.

It is worth mentioning that most studies reporting hormonal changes presented their conclusions while only considering the statistical significance in response to stressors. There was no discussion as to whether these modifications were clinically significant or if they represented meaningful changes, such as modifying values out of their reference ranges. Only Henning and cols. ( 32 ) discussed their testosterone results with consideration of the normal lower limits and found that approximately 74% of the 23 participants presented values equivalent to hypogonadism. Future studies should use both strategies of discussion: statistical and clinical significance.

Higher myoglobin serum levels probably relate to muscle cell damage since it is the result of the release of muscle cell contents into the plasma after cell disruption ( 3 – 5 ). Only one publication measured this biomarker of cellular injury in relation to the triad of interventions experienced from FTX. It should be noted, however, that other biomarkers of cellular injury, such as creatin-kinase (CK), alanine transaminase (ALT), and aspartate transaminase (AST), are present in studies that do not simultaneously apply the three different interventions ( 3 , 4 ). Surprisingly, variations in biomarkers of oxidative stress are scarcely studied in relation to FTX. Biomarkers of oxidative stress could serve to measure the physical condition of a participant to evaluate their physical readiness for military operational field training. This could exclude participants with detectable signs of overreaching/overtraining prior to FTX. It could also improve training schedules and diets, aimed at better military preparedness by avoiding injuries and optimizing physiological adaptation pathways, which is important considering the endurance features of military field training exercises (FTX) and the insufficient time of preparation of these soldiers. These biomarkers’ assessments would also be useful as posttraining recovery protocols to monitor the individuals’ process of physical repair. We also suggest a search for the connection between changes in hormone levels changes, inflammatory mediators and biomarkers of oxidative stress to better comprehend the adaptive pathways to the simultaneous combination of interventions.

Our review also identified a lack of inclusion of female military personnel in past studies. Consequently, there is little information on the physiological variations experienced by women during an FTX, especially considering the simultaneous application of these three interventions, which is important to preserve the simulation conditions for actual military operations. Considering the gradual yet steady increase in the recruitment of women into the armed forces, future studies should consider hormonal and biochemical responses in women in relation to FTX. The absence of data on women was not related to our literature search. While our review was limited to searches in only two databases (PubMed and Scopus), both are highly representative of our fields of interest to the present studies. The frequent absences of experimental details on the characteristics of FTX in some analyzed articles made it difficult to determine the influencing factors that could explain some of the conflicting results. Nevertheless, the generally low risk of bias of the articles reviewed suggests that the inclusion/exclusion screen was of high quality. Only performance bias remained a high-risk parameter, which can be related to the difficulties for researchers in this area in creating the ideal conditions for a double-blind study. Furthermore, most studies were conducted during an actual FTX (and not simulated interventions), which suggests that all participants experienced similar stress conditions. Although most studies did not report the actual temperatures, the descriptions of the FTX did not include thermal factors which suggests similar weather conditions.

In conclusion, the joint intervention comprising prolonged physical effort, food restriction, and sleep deprivation combined during military field training exercises provoked a decrease in levels of thyroid and anabolic hormones, except for growth hormone, which increased. The other measured hormones--cortisol, TSH and LH--showed a less consistent response to FTX. The data on inflammatory mediators and biomarkers for cellular injury were insufficient to draw conclusions. Both variables increased in the few studies retrieved on the topic, which could explain the impaired physical performance due to FTX, jeopardizing the operational readiness of personnel in combat scenarios and putting individuals at risk for physical harm. Finally, this review found a lack of data about the modifications of biomarkers of oxidative stress after FTX, which prevented this research from drawing conclusions on a few physiological pathways that remain obscure to the present date. Another important fact, identified by the present review, is the lack of studies following hormonal and biochemical responses in female military units, which encourages future studies to bridge the gap.
